# Plasmid Transfer in the Ocean – A Case Study from the Roseobacter Group

**DOI:** 10.3389/fmicb.2017.01350

**Published:** 2017-07-18

**Authors:** Jörn Petersen, Irene Wagner-Döbler

**Affiliations:** ^1^Research Group Plasmids and Protists, Leibniz-Institute DSMZ – German Collection of Microorganisms and Cell Cultures Braunschweig, Germany; ^2^Research Group Microbial Communication, Helmholtz – Center for Infection Research Braunschweig, Germany

**Keywords:** plasmid synteny, type IV secretion systems, conjugation, horizontal gene transfer, evolution

## Abstract

Plasmid mediated horizontal gene transfer (HGT) has been speculated to be one of the prime mechanisms for the adaptation of roseobacters (*Rhodobacteraceae*) to their ecological niches in the marine habitat. Their plasmids contain ecologically crucial functional modules of up to ∼40-kb in size, e.g., for aerobic anoxygenic photosynthesis, flagellar formation and the biosynthesis of the antibiotic tropodithietic acid. Furthermore, the widely present type four secretion system (T4SS) of roseobacters has been shown to mediate conjugation across genus barriers, albeit in the laboratory. Here we discovered that *Confluentimicrobium naphthalenivorans* NS6^T^, a tidal flat bacterium isolated in Korea, carries a 185-kb plasmid, which exhibits a long-range synteny with the conjugative 126-kb plasmid of *Dinoroseobacter shibae* DFL12^T^. Both replicons are stably maintained by RepABC operons of the same compatibility group (-2) and they harbor a homologous T4SS. Principal component analysis of the codon usage shows a large similarity between the two plasmids, while the chromosomes are very distinct, showing that neither of the two bacterial species represents the original host of those RepABC-2 type plasmids. The two species do not share a common habitat today and they are phylogenetically only distantly related. Our finding demonstrates the first clear-cut evidence for conjugational plasmid transfer across biogeographical and phylogenetic barriers in *Rhodobacteraceae* and documents the importance of conjugative HGT in the ocean.

## Introduction

Horizontal gene transfer (HGT) dominates prokaryotic evolution (Koonin, 2016). In addition to plasmid transfer by conjugation, two other mechanisms of HGT are known in the ocean, namely, transformation via direct uptake of DNA and transduction by phages or gene transfer agents (GTAs). Plasmids have an important role because of the large amount of genetic material that can be transferred in a single conjugation event. Thus, uptake of a plasmid may allow the host to colonize an entirely new niche. For example, the virulence plasmid of *Shigella* converts a harmless *Escherichia coli* commensal into a deadly pathogen (The et al., 2016). The spread of antibiotic resistance plasmids across the globe powered by the selective pressure exerted by antibiotic misuse and overuse, represents an unsolved challenge to our health-care system (Palmer et al., 2010; Perry and Wright, 2013). The rapidity of this process is exemplified by the spread of resistance against colistin, the antibiotic of last resort, encoded by the plasmid-located *mrc-1* gene. It was first discovered in November 2015 in commensal *E. coli* isolates from food animals in China (Liu et al., 2016) and six month later the first colistin-resistant *E. coli* strain was isolated from a patient in Pennsylvania, United States (McGann et al., 2016).

Little is known about HGT by conjugation in the ocean. Since it requires close physical contact between donor and recipient, it is thought to be restricted to hot spots like aggregates or surfaces, where bacteria form biofilms (Thibault and Top, 2016), have high cell densities and are metabolically active (Sobecky and Hazen, 2009). Studies until now have been restricted to microcosms (Aminov, 2011).

Roseobacters, a marine subgroup of the *Rhodobacteraceae*, carry up to 12 plasmids per cell (Pradella et al., 2010). The maintenance of these low copy number plasmids is ensured by a characteristic tripartite module comprising a replicase initiating their duplication at the origin of replication (*ori*) and two partitioning genes that mediate the coordinated anchorage of the replicons at the cell poles during cell division (Petersen, 2011). The most abundant plasmid type of roseobacters is represented by RepABC-type replicons comprising at least nine different compatibility groups that ensure their stable coexistence within the same cell (Petersen et al., 2009). The genetic functions encoded on Roseobacter plasmids are potential drivers of adaptation to ecological niches in the ocean, e.g., aerobic anoxygenic photosynthesis, synthesis of the antibiotic tropodithietic acid, biofilm formation, or killing of dinoflagellate cells (Petersen et al., 2013; Wang et al., 2015). Many of those plasmids carry type IV secretion systems (T4SS) (Chandran Darbari and Waksman, 2015), suggesting that they might be conjugative. Indeed it could be demonstrated that two syntenic sister plasmids of *Dinoroseobacter shibae* DFL12^T^, which originate from a plasmid duplication and the recruitment of a novel RepABC-type replication module (Wagner-Döbler et al., 2010), can be transferred to *Phaeobacter inhibens* DSM 17395 by conjugation *in vitro* (Patzelt et al., 2016). *Dinoroseobacter* is among the deepest branching genera within the Roseobacter group, while *Phaeobacter* is located in the most distant subclade (Simon et al., 2017), showing that these plasmids have a very broad host range in *Rhodobacteraceae*.

Here we provide insights into the evolutionary history of the conjugative 126-kb plasmid of *D. shibae.* We show that a syntenic plasmid is naturally present in two phylogenetically distant species of the Roseobacter group, i.e., *Confluentimicrobium naphthalenivorans* and *Roseovarius indicus*. Codon usage analysis shows that the plasmids have been obtained from different hosts, and thus have probably been transferred multiple times – they have “tramped” through the phylogenetic tree of *Rhodobacteraceae*. To the best of our knowledge, these observations provide the first example for HGT by conjugation in the ocean.

## Results and Discussion

### Detection of Syntenic Plasmids

**FIGURE 1:**
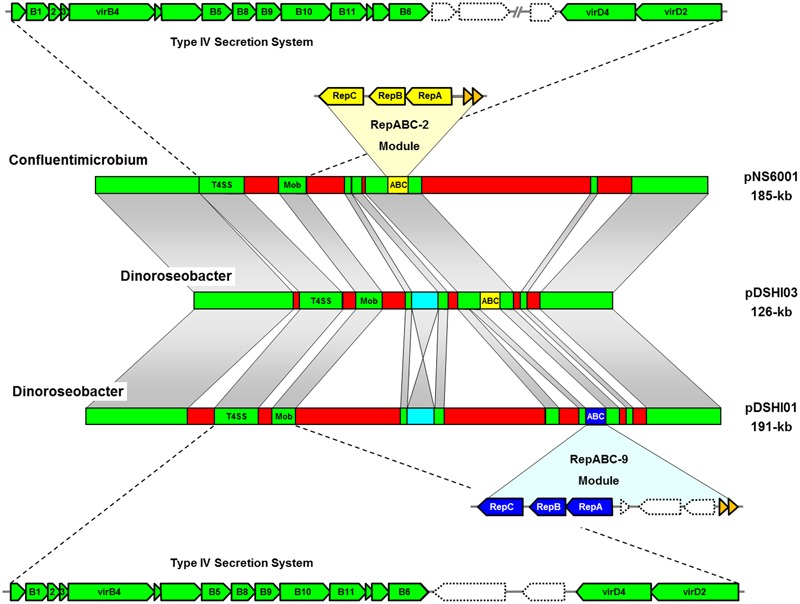
Synteny plot of the 185-kb RepABC-2 type plasmid from *Confluentimicrobium naphthalenivorans* NS6^T^ (pNS6001) and the two sister plasmids from *Dinoroseobacter shibae* DFL12^T^ (pDSHI01, pDSHI03). Long-range homologies with conserved genes are shown with fading gray bars and syntenic regions including the type IV secretion systems (T4SS) for plasmid conjugation are shown in green. Plasmid replication systems of the RepABC-2 and RepABC-9 type are highlighted in yellow and blue, respectively. Adjacent toxin/antitoxin systems are shown in orange. A *D. shibae* specific insertion is highlighted in turquoise. Homologous genes were determined by comparative TBLASTN analyses (see Supplementary Table S1).

The improvement of sequencing technologies in the last decade resulted in an exponential increase of completely deciphered bacterial genomes with more than 400 *Rhodobacteraceae* currently deposited in public data bases. We used the crucial replicases of the two sister plasmids from *D. shibae* for BLASTP searches and could identify a 185-kb plasmid from *Confluentimicrobium naphthalenivorans* NS6^T^ (pNS6001; Jeong et al., 2015) that exhibits a long range synteny with the 126-kb plasmid pDSHI03 (**Figure 1**). Both replicons share the type IV secretion system (T4SS), toxin/antitoxin modules and a RepABC-2 type replication operon for plasmid maintenance (yellow), which has been replaced in the 191-kb sister plasmid pDSHI01 by a compatible RepABC-9 type equivalent (blue). Cytochrome *c* biosynthesis and heavy metal detoxification genes are the most conspicuous shared life style determinants of these extrachromosomal replicons (ECRs; Supplementary Table S1). Comprehensive TBLASTN comparisons revealed a protein sequence identity between 92 and 100% for most conserved genes, and the differences reflect their individual evolutionary history in the respective host cell. For example, the selective pressure on the functional genes is indirectly documented by an about 10% lower conservation of several hypothetical proteins, thus providing evidence that the plasmids pNS6001 and pDSHI03 were not recently transferred. However, 74% of the genes from pDSHI03 are shared with the homologous RepABC-2 plasmid from *Confluentimicrobium* (Supplementary Table S1). The structural backbone of both plasmids is absolutely conserved apart from some unique regions, such as the large 66-kb insertion in pNS6001 (**Figure 1**). The degree of sequence conservation even exceeds that of the stably coexisting 126-kb and 191-kb sister plasmids of *D. shibae* that harbor an inverted InDel (see below), but exhibit different compatible replication modules of the RepABC-2 and -9 type representing the ‘heart of a plasmid’ (Wagner-Döbler et al., 2010).

Our BLASTP searches also revealed the presence of a 135-kb contig with RepABC-2 replication module in the draft genome of *Roseovarius indicus* EhC03 (Rosana et al., 2016) that likely represents a second syntenic plasmid. The contig is less conserved than pNS6001, but still contains 45% of the genes from pDSHI03 including the T4SS (Supplementary Table S2). Systematic TBLASTN based comparisons of the respective replicons showed a slightly lower degree of protein conservation. The benefit of reciprocal BLAST analyses of conserved DNA modules is exemplified by the detection of four pseudogenes in *D. shibae*. The SAM-dependent methyltransferase from *R. indicus* (OAO03639.1) and *C. naphthalenivorans* (WP_054540530.1) matches with two annotated genes of the 126-kb plasmid of *D. shibae* (Dshi_4003 [WP_050757929.1], Dshi_4008 [WP_012187351.1]) that are separated by a 6-kb fragment containing a type III restriction endonuclease and integrases (Dshi_4004 to Dshi_4007; highlighted in turquoise, **Figure 1**). This arrangement suggests that the functional gene in *Dinoroseobacter* was inactivated by a transposition event (Supplementary Table S2). A homologous but inverted insertion is present on the 191-kb plasmid (Dshi_3695 to Dshi_3698) resulting in two annotated SAM-dependent methyltransferase pseudogenes (Dshi_3694 [WP_012187109.1], Dshi_3699 [WP_012187104.1]), which is illustrated by the hourglass shaped syntenic area between the sister plasmids in **Figure 1**. This case example documents that a thorough comparison of closely related sequences helps to improve the manual genome annotation of model organisms such as *D. shibae* DFL12^T^ (Wagner-Döbler et al., 2010).

### RpoB Phylogeny as a First Proxy for the Taxonomic Positioning

**FIGURE 2:**
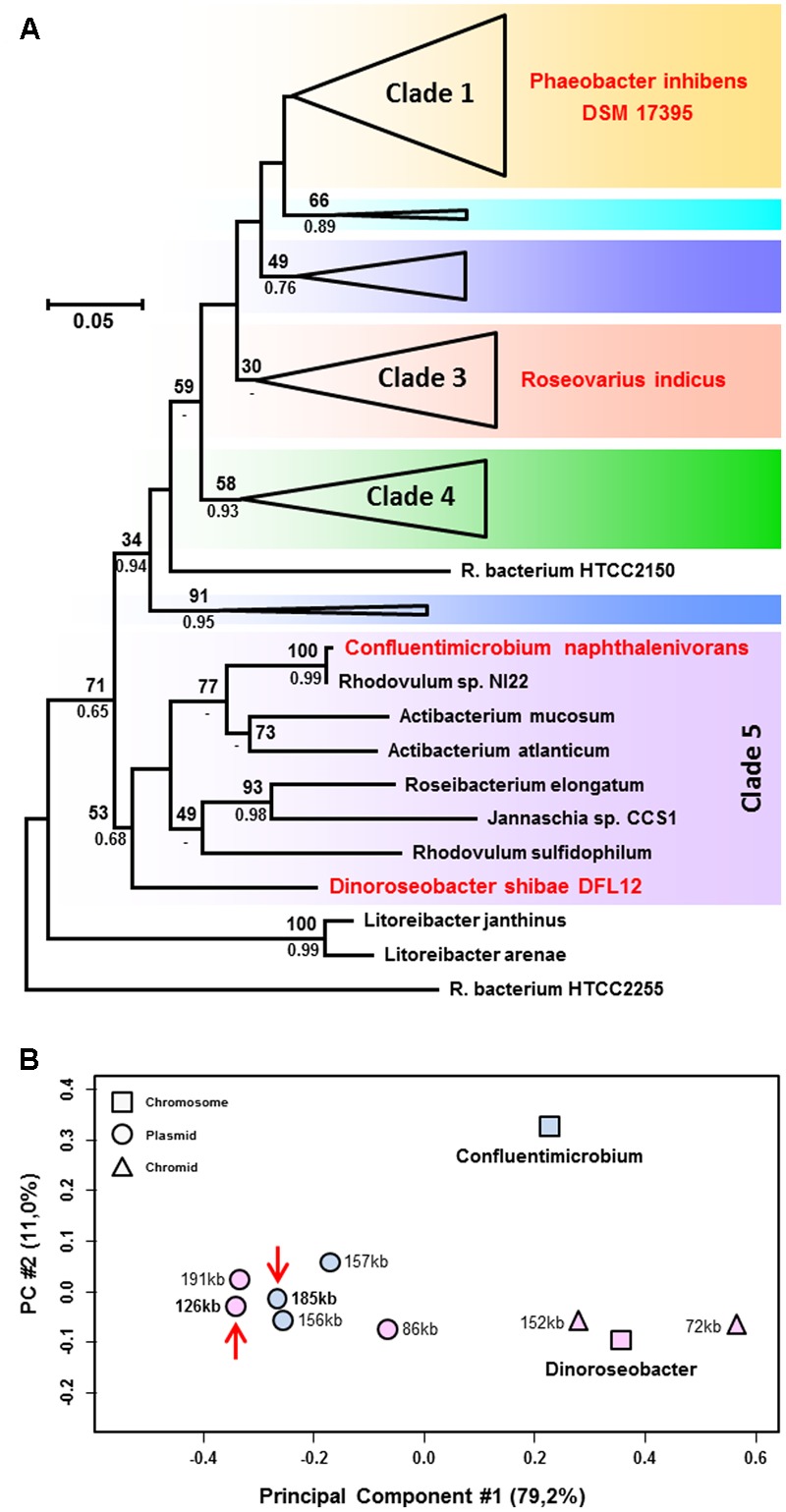
**(A)** Phylogenetic RpoB analysis from roseobacters based on 69 sequences and 1374 amino acid positions. Strains harboring natural syntenic RepABC-2 type plasmids such as *Dinoroseobacter shibae* and *Confluentimicrobium naphthalenivorans* or served as a recipient for plasmid conjugation (*Phaeobacter inhibens*) are highlighted in red. The color code of the seven subtrees (clades) corresponds to a phylogenomic analysis presented by Michael et al. (2016); the complete tree is shown in **Supplementary Figure S1**. **(B)** Principal component analysis of the relative synonymous codon usage of replicons from *D. shibae* and *C. naphthalenivorans*. Chromosomes, chromids and plasmids are shown by squares, triangles and circles, respectively. The positioning of the syntenic RepABC-2 type plasmids is indicated by red arrows.

A phylogenetic RaxML tree of the RNA polymerase beta subunit (RpoB) from 69 Roseobacter strains was calculated in order to reveal the relationships of the host cells of the syntenic RepABC-2 type plasmids (**Supplementary Figure S1**). The taxon sampling included the natural hosts *D. shibae, C. naphtahalenivorans* as well as *R. indicus* and largely corresponded to that of a recent phylogenomic analysis of 65 Roseobacter genomes (Michael et al., 2016). Our single gene phylogeny recovered all seven clades as monophyletic groups and many subtrees were even supported with a solid bootstrap proportion (BP). Furthermore, it also mirrors the branching pattern of a phylogenomic analysis based on 44 roseobacters with a different taxon sampling including three single-cell genomes from an uncultured streamlined lineage from the ocean surface (Luo et al., 2014). This outcome documents that the phylogenetic position of novel *Rhodobacteraceae* genomes can rapidly be estimated by a single gene RpoB analysis, which is based on 1374 amino acid (aa) alignment positions only, thus serving as a first proxy for complex phylogenomic studies with more than 200,000 aa positions (Case et al., 2007; Michael et al., 2016). The broader taxon sampling of eight genome sequenced strains from clade 5 (former analyses were restricted to two or three strains in this particular subgroup; Luo et al., 2014; Michael et al., 2016; Simon et al., 2017) recovered a very close branching of *Confluentimicrobium* with *Rhodovulum* sp. NI22 and a sistergroup relationship with the genus *Actibacterium* (77% BP; **Figure 2A**). The basal position of *Dinoroseobacter* shows a considerable evolutionary distance to *Confluentimicrobium.* The absence of syntenic RepABC-2 replicons and even plasmids of the same compatibility group from the remaining six Roseobacter strains in clade 5 is in agreement with a horizontal recruitment by *D. shibae* and *C. naphthalenivorans* from taxa outside of clade 5, a hypothesis that is based on the distinct codon usage (CU; see below) and thus more plausible than the alternative explanation of a common ancestry followed by differential plasmid losses. The phylogenetic position of *Roseovarius indicus* in clade 3 indicates that the syntenic RepABC-2 plasmids (**Figure 1**; Supplementary Tables S1, S2) are migrating within the Roseobacter group. This prediction is supported by the experimental conjugation of pDSHI03 into *P. inhibens* DSM 17395 (Patzelt et al., 2016) thereby connecting clades 1 and 5 (**Figure 2A**). Syntenic RepABC-2 plasmid have thus been experimentally transferred between the most distant clades, and can naturally be detected in members of clade 5 and 3, thus documenting independent events of lateral gene transfer in the ocean.

### Codon Usage Analysis of *Confluentimicrobium* and *Dinoroseobacter* Replicons

The relative synonymous codon usage (RSCU) of the chromosome and ECRs from *D. shibae* and *C. naphthalenivorans* was analyzed using methods described previously (Petersen et al., 2013) and a plot of the averaged RSCU of protein-coding genes of all ten replicons is shown in **Figure 2B**. The principal component (PC) analysis confirmed the classification of *D. shibae*’s 152-kb and 72-kb ECRs as chromids, which exhibit a RSCU comparable to that of the chromosome (Harrison et al., 2010), whereas the three more distinct replicons of 86-kb, 126-kb and 191-kb represent authentic plasmids. The RSCU analysis of all *Confluentimicrobium* replicons showed a distinct localization of the three ECRs compared with the chromosome providing evidence that they also represent typical plasmids. This conclusion is independently supported by the presence of structurally conserved T4SS on the chromosome and all three ECRs (Supplementary Table S3) that contain RepABC operons of different compatibility groups (pNS6001 [185-kb, RepABC-2], pNS6002 [157-kb, RepABC-9], pNS6003 [156-kb, RepABC-5]). RepABC modules represent the most frequently mobilized plasmid type among the four characteristic replication systems of Roseobacter ECRs (RepA, RepB, DnaA-like, RepABC; Petersen et al., 2013). Based on the detection of a RepABC-9 operon on another replicon of *Confluentimicrobium*, the 157-kb plasmid pNS6002, which is homologous and functionally equivalent to that of the 191-kb sister plasmid pDSHI01 from *D. shibae* (blue module, **Figure 1**), we compared both plasmids via BLASTN, but observed only a limited structural conservation for the replication module and the generally conserved T4SS. However, the presence of a co-existing RepABC-2 and -9 plasmid pair in two Roseobacter species reflects a stable co-evolution of these ECRs. The close grouping of the syntenic 126-kb pDSHI03 and 185-kb pNS6001 plasmids in the RSCU, which is highlighted with red arrows in **Figure 2B**, suggests a common origin of both replicons. The large distance of both replicons to their respective chromosomes unequivocally documents that neither *D. shibae* nor *Confluentimicrobium* is the genuine host cell of this conjugative RepABC-2 type plasmid.

### Origin and Habitat of Roseobacter Isolates

*Dinoroseobacter shibae* DFL12^T^ was isolated from a culture of the dinoflagellate *Prorocentrum lima* maintained at the Biological Research Station of the Alfred-Wegener-Institute for Polar Research (AWI) at Helgoland in the North Sea (Biebl et al., 2005). The algal culture was a gift from the Toralla Marine Science Station (ECIMAT) of the University of Vigo, Spain, where the dinoflagellate had been isolated from the North Atlantic Ocean. *C. naphtalenivorans* NS6^T^ was isolated from heavily polluted tidal flat sediments in the South Sea, a part of the Pacific Ocean adjacent to South Korea (Jeong et al., 2015), at a distance of roughly 8000 km to Vigo, Spain. Thus, today those two species do not share a similar habitat or location. *Roseovarius indicus* EhC03 was isolated from a culture of the coccolithophore alga *Emiliania huxleyi* M217 maintained at the Plymouth Algal Collection, United Kingdom. The algae had originally been isolated from surface water of the South Pacific (NCBI BioSample SAMN04965936; Rosana et al., 2016). The synteny of the *R. indicus* RepABC-2 plasmid with pDSHI03 of *D. shibae* is interesting and it could be speculated if those plasmids share conserved traits that are beneficial for the association of roseobacters with eukaryotic microalgae from phylogenetically distant phyla, e.g., haptophytes (*Emiliania huxleyi*) and dinoflagellates (*Prorocentrum lima*).

## Perspective

The discovery of long-range synteny between conjugative plasmids in species from the Roseobacter group which are phylogenetically distant and geographically separated by 1000s of kilometers today provides the first proof for independent events of conjugation in the ocean that were stably maintained in the respective species. Plasmid mediated HGT likely plays an important role for the evolution of roseobacters.

## Author Contributions

JP conceived the study and performed the analyses; JP and IWD wrote the manuscript.

## Conflict of Interest Statement

The authors declare that the research was conducted in the absence of any commercial or financial relationships that could be construed as a potential conflict of interest.

## Supplementary Material

FIGURE S1 Phylogenetic Maximum Likelihood tree of the RNA-polymerase beta subunit from roseobacters (RaxML, LG + F + 4Γ). The RpoB analysis was based on 69 sequences and 1374 amino acid positions. The statistical support for the internal nodes of the RaxML tree was determined by 100 bootstrap replicates (BR) and values > 30% are shown (upper value). Posterior probabilities were calculated with PhyloBayes v3 (CATGTR + 4Γ; lower value). Strains that harbor syntenic RepABC-2 type plasmids (*D. shibae, C. naphthalenivorans, R. indicus*) or served as conjugational recipient (*P. inhibens*) are highlighted in red. The color code of the seven clades corresponds to a phylogenomic analysis presented by Michael et al. (2016).
