# Supplementary figures and images for: Plasmid Transfer in the Ocean – A Case Study from the Roseobacter Group
Source: Front Microbiol. 2017 Jul 18;8:1350. doi: 10.3389/fmicb.2017.01350 (PMC5513947; doi:10.3389/fmicb.2017.01350)

Fig. S1

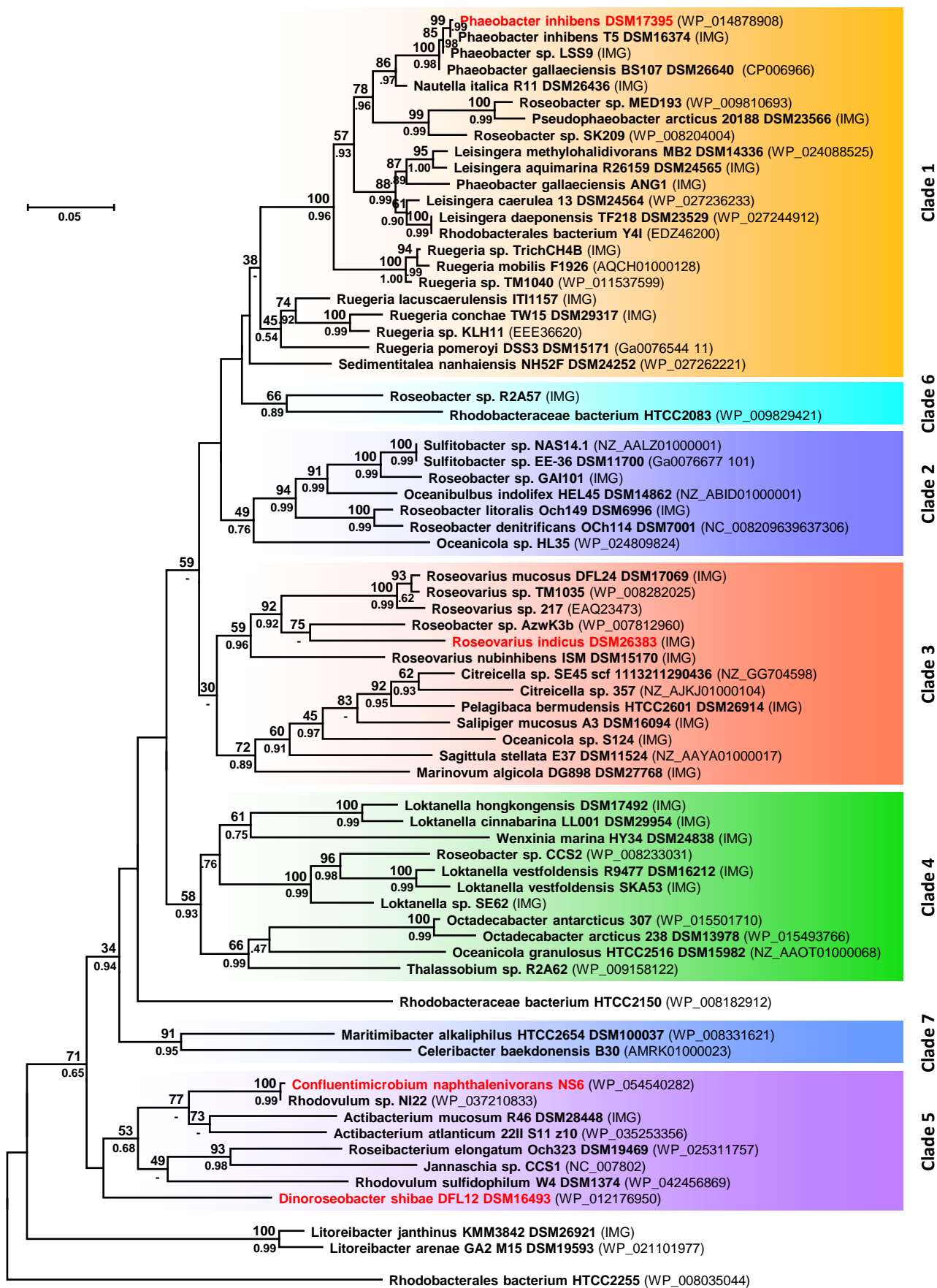

Supplement: FIGURE S1 — Phylogenetic Maximum Likelihood tree of the RNA-polymerase beta subunit from roseobacters (RaxML, LG + F + 4Γ). The RpoB analysis was based on 69 sequences and 1374 amino acid positions. The statistical support for the internal nodes of the RaxML tree was determined by 100 bootstrap replicates (BR) and values > 30% are shown (upper value). Posterior probabilities were calculated with PhyloBayes v3 (CATGTR + 4Γ; lower value). Strains that harbor syntenic RepABC-2 type plasmids (D. shibae, C. naphthalenivorans, R. indicus) or served as conjugational recipient (P. inhibens) are highlighted in red. The color code of the seven clades corresponds to a phylogenomic analysis presented by Michael et al. (2016). [file Image_1.PDF]

# RpoB, PhyloBayes, 69 taxa (CATGTR+4Γ)

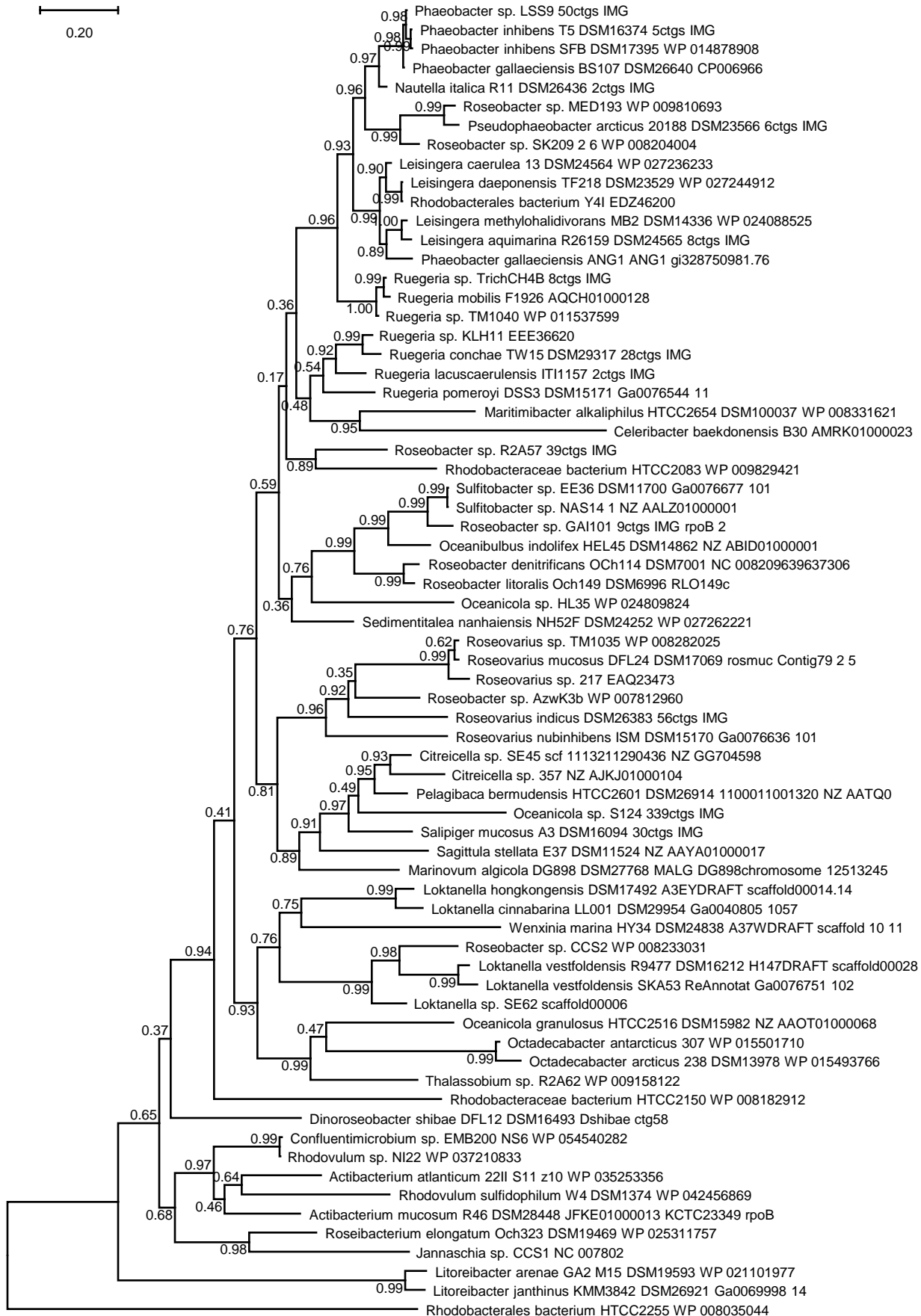

Supplement: Supplementary file 2 [file Image_2.PDF]
